# Genetic characteristics and pathogenesis of H5 low pathogenic avian influenza viruses from wild birds and domestic ducks in South Korea

**DOI:** 10.1038/s41598-020-68720-w

**Published:** 2020-07-22

**Authors:** Yu-Na Lee, Dong-Hun Lee, Sun-Ha Cheon, Yu-Ri Park, Yoon-Gi Baek, Young-Jae Si, Soo-Jeong Kye, Eun-Kyoung Lee, Gyeong-Beom Heo, You-Chan Bae, Myoung-Heon Lee, Youn-Jeong Lee

**Affiliations:** 1 Avian Influenza Research and Diagnostic Division, Animal and Plant Quarantine Agency, 177 Hyeoksin 8-ro, Gimcheon-si, Gyeongsangbuk-do 39660 Republic of Korea; 2 Department of Pathobiology and Veterinary Science, University of Connecticut, 61 North Eagleville Road, Unit 3089, Storrs, CT USA; 3 Avian Disease Division, Animal and Plant Quarantine Agency, 177 Hyeoksin 8-ro, Gimcheon-si, Gyeongsangbuk-do 39660 Republic of Korea

**Keywords:** Microbiology, Molecular biology

## Abstract

H5 and H7 subtypes of low pathogenic avian influenza viruses (LPAIVs) can mutate to highly pathogenic forms and are therefore subject to stringent controls. We characterized H5 LPAIVs isolated from wild-bird habitats and duck farms in South Korea from 2010 to 2017. Through nationwide active surveillance for AIVs, 59 H5 LPAIVs were isolated from wild-bird habitats (a mean annual rate of 5.3% of AIV isolations). In 2015, one LPAI H5N3 strain was isolated on a duck farm. Phylogenetic analysis revealed that the hemagglutinin (HA) gene of H5 isolates belonged to the Eurasian lineage, classified into three subgroups (HA-II, HA-III, and HA-IV). The H5 LPAIVs of the HA-III and HA-IV subgroups appeared in 2015 and 2017 in unusually high proportions (13.1% and 14.4%, respectively). In gene-constellation analysis, H5 LPAIVs isolated from 2015 to 2017 constituted ≥ 35 distinct genotypes, representing high levels of genetic diversity. Representative strains of three HA subgroups replicated restrictively in specific-pathogen-free chickens. Among the 11 isolates that were tested, 10 infected and replicated in mice without prior adaptation. The frequency of recent H5 LPAIV isolates with high genetic diversity indicates the importance of continued surveillance in both wild birds and poultry to monitor genetic and pathobiological changes.

## Introduction

Influenza A viruses (IAVs) are single-stranded, negative-sense RNA viruses with eight genomic segments. Wild birds, especially migratory waterfowl, are considered to be the natural reservoir for IAVs and maintain a huge viral genetic pool^1^. Avian influenza viruses (AIVs) have been identified to date with surface proteins of the 16 hemagglutinin (H1–16) and nine neuraminidase (N1–9) subtypes^2^. Low pathogenic avian influenza (LPAI) is a natural enteric infection of waterfowl that is usually subclinical. Through migration, waterfowl carrying AIVs contribute to spillover of diverse viruses into domestic poultry along the flyway. Infection of gallinaceous poultry with LPAI H5 and H7 viruses has the potential, by viral evolution, to result in highly pathogenic avian influenza (HPAI) through mechanisms that enhance the cleavability of hemagglutinin (HA) glycoprotein by insertion or substitution of multiple basic amino acids at the cleavage site^3^, or by non-homologous recombination with other viral genes or the host genome^4–6^. Outbreaks of H5 and H7 HPAI viruses (HPAIVs) have caused high mortality in gallinaceous poultry, resulting in serious economic losses in the poultry industry worldwide^7^.

To date, several independent LPAI virus (LPAIV) to HPAIV conversion events have been documented since the first H5 HPAI virus was identified from chicken farms in Scotland in 1959^2,8,9,10^. In April 1983, an H5N2 LPAIV was detected at a chicken farm in Pennsylvania, USA; this virus may have been derived from LPAIVs harbored by wild waterfowl^11,12^. Half a year later, this virus had mutated into a highly pathogenic variant, causing the death or culling of > 17 million birds. From March to December 1999, 199 outbreaks of H7N1 LPAI were detected in northern Italy^13,14^. Despite control measures, H7N1 HPAI infection was detected in December 1999 and spread to the industrial poultry population with a total of 413 outbreaks, causing the deaths of > 16 million birds^15^.

A nationwide surveillance strategy in South Korea has been designed to focus on early detection and response to H5 or H7 LPAI and HPAI. In the present study, we characterized 48 H5 LPAI isolates derived from migratory waterfowl and domestic ducks in South Korea from 2010 to 2017. To better understand the evolution and genetic diversity of these H5 LPAIVs, we sequenced the full-length genomes of the isolates and analyzed their genetic characteristics. Moreover, we evaluated their replication and pathogenic potential in chickens and mice.

## Results

### Isolation of H5 LPAIVs in South Korea

From 2010 to 2017, a total of 770 IAVs were isolated from wild-bird habitats (Table 1). Among the 59 H5 LPAIVs that were isolated, H5N3 viruses were the predominant subtype (40/59, 67.8%), followed by H5N2 (16/59, 27.1%) and H5N9 (2/59, 3.4%). The mean annual proportion of isolated viruses that were H5 LPAIVs was 5.3%, with a range of 0–14.4%. In 2015, H5 LPAIVs were 19 of the 145 isolated viruses (13.1%), and in 2017, H5 LPAIVs were 26 of the 180 viruses isolated from wild-bird habitats (14.4%).

**Table 1:** Numbers of avian influenza viruses (AIVs) isolated from wild bird habitats and duck farms in South Korea between 2010 and 2017.

Target	Year							
	2010	2011	2012	2013	2014	2015	2016	2017
**Wild birds**								
Total no. of samples	6,789	7,156	7,889	10,029	13,228	19,533	9,538	15,001
No. of AIV-positive samples (prevalence^a^)	56 (0.8%)	48 (0.7%)	42 (0.5%)	22 (0.2%)	137 (1.0%)	145 (0.7%)	140 (1.5%)	180 (1.2%)
No. of H5N2 LPAIVs	1							15
No. of H5N3 LPAIVs			1		3	17	8	11
No. of H5N9 LPAIVs						2		
No. of mixed H5 LPAIVs			1					
Subtotal (proportion^b^)	1 (1.8%)	0	2 (4.8%)	0	3 (2.2%)	19 (13.1%)	8 (5.7%)	26 (14.4%)
**Duck farms**								
Total no. of samples	–	27,157	23,000	123,342	42,044	203,312	182,376	152,304
No. of AIV-positive samples (prevalence^a^)	–	20 (0.07%)	35 (0.15%)	102 (0.08%)	54 (0.13%)	55 (0.03%)	14 (0.01%)	15 (0.01%)
No. of H5N3 LPAIVs						1		
Subtotal (proportion^b^)	0	0	0	0	0	1 (1.8%)	0	0

^a^No. of AIV-positive samples/total no. of samples (%).

^b^No. of H5 LPAIVs/no. of AIV-positive samples (%).

LPAIV, low pathogenic AIV.

In the active domestic-duck surveillance program, diagnostic testing for IAVs was performed for carcasses and for live birds prior to shipment to the slaughterhouse. From 2011 to 2017, 295 IAVs were isolated from 753,535 samples from domestic-duck farms, and the mean annual proportion of samples that were IAVs was 0.07%, with a range of 0.01–0.15%. In 2015, among 55 IAVs, one was an H5N3 LPAIV.

### Molecular characteristics of H5 LPAIVs

Representative isolates (*n* = 48; Supplementary Table 1) from among the 60 H5 LPAIVs were selected for molecular characterization, excluding redundant viruses from an individual sampling point. All HA proteins of the characterized viruses carried the cleavage-site motif PQRETR/GLF (Table 2), indicating low pathogenicity in chickens^16^. The amino acids at positions 222 and 224 (numbering based on H5 A/Vietnam/1203/2004) of the receptor-binding sites were glutamine and glycine, respectively, indicating a preference for the avian-like receptor rather than the human-like receptor. However, in all isolates the HA amino acid substitution T156A was present, which increases affinity for the human-like sialic acid receptor^17,18^. The E627K substitution in the polymerase basic protein 2 (PB2), which has a prominent role in mammalian adaptation^19^, was not detected in any of the H5 viruses. Only one isolate, A/spot-billed duck/Korea/H51/2017 (H5N2) had the PB2 substitution D701N, which facilitates viral polymerase activity in mammals^20,21^. Six substitutions in PB2 (L89V, G309D, T339K, R477G, I495V, and A676T), which can compensate for the effect of the E627K substitution in mice^22^, were identified in most H5 LPAI viruses. Nine out of 48 isolates had the N66S substitution in PB1-F2, which has been shown to increase virulence by inhibiting the early interferon response in mice^23,24^. Signature mammalian-adaptive mutations in other proteins were also identified in some of the representative isolates, such as S409N and K615R in polymerase acidic protein (PA)^25^, V15I^26^ in matrix protein 1 (M1), and P42S in non-structural protein 1 (NS1)^27^.

**Table 2:** Amino acid sequences and substitutions in H5 low pathogenic avian influenza viruses (LPAIVs) isolated from wild birds in South Korea between 2010 and 2017.

Virus name	HA^a^				M1	NS1	PB2								PB1-F2	PA	
	Cleavage site	T156A	Q222L	G224S	V15I	P42S	L89V	G309D	T339K	R477G	I495V	E627K	A676T	D701N	N66S	S409N	K615R
A40-1/12	PQRETR↓GLF	A	Q	G	V	A	V	D	K	G	V	E	T	D	S	S	K
H598/14	PQRETR↓GLF	A	Q	G	V	S	V	D	K	G	V	E	T	D	S	S	K
H1862/14	PQRETR↓GLF	A	Q	G	V	S	V	D	K	G	V	E	T	D	N	S	K
H2016/14	PQRETR↓GLF	A	Q	G	V	S	V	D	K	G	V	E	T	D	N	S	K
H2193/15	PQRETR↓GLF	A	Q	G	V	S	V	D	K	G	V	E	T	D	N	S	K
H2262/15	PQRETR↓GLF	A	Q	G	V	S	V	D	K	G	V	E	T	D	N	S	K
H2512/15	PQRETR↓GLF	A	Q	G	I	S	V	D	K	G	V	E	A	D	N	S	K
H3328/15	PQRETR↓GLF	A	Q	G	V	S	V	D	K	G	V	E	T	D	N	S	K
H3373/15	PQRETR↓GLF	A	Q	G	V	S	V	D	K	G	V	E	T	D	N	S	K
H2292/15	PQRETR↓GLF	A	Q	G	V	S	V	D	K	G	V	E	T	D	N	S	K
H2318/15	PQRETR↓GLF	A	Q	G	V	S	V	D	K	G	V	E	T	D	N	S	K
H3135/15	PQRETR↓GLF	A	Q	G	V	S	V	D	K	G	V	E	T	D	N	S	K
H3173/15	PQRETR↓GLF	A	Q	G	V	S	V	D	K	G	V	E	T	D	N	S	K
H3306/15	PQRETR↓GLF	A	Q	G	V	A	V	D	K	G	V	E	T	D	N	S	K
H3316/15	PQRETR↓GLF	A	Q	G	V	S	V	D	K	G	V	E	T	D	N	S	K
H3334/15	PQRETR↓GLF	A	Q	G	V	S	V	D	K	G	V	E	T	D	N	S	K
H3422/15	PQRETR↓GLF	A	Q	G	V	S	V	D	K	G	V	E	T	D	N	S	K
H3473/15	PQRETR↓GLF	A	Q	G	V	S	V	D	K	G	V	E	T	D	N	S	K
H50/16	PQRETR↓GLF	A	Q	G	V	S	V	D	K	G	V	E	T	D	N	S	K
H95-4/16	PQRETR↓GLF	A	Q	G	V	S	V	D	K	G	V	E	T	D	N	S	K
H96-1/16	PQRETR↓GLF	A	Q	G	V	S	V	D	K	G	V	E	T	D	N	S	K
H125-4/16	PQRETR↓GLF	A	Q	G	V	S	V	D	K	G	V	E	T	D	N	S	K
H886/16	PQRETR↓GLF	A	Q	G	V	S	V	D	K	G	V	E	T	D	N	S	K
H1069-3/17	PQRETR↓GLF	A	Q	G	V	S	V	D	K	G	V	E	T	D	N	S	K
H189-1/16	PQRETR↓GLF	A	Q	G	V	S	V	D	K	G	V	E	T	D	N	S	K
A44-1-3/16	PQRETR↓GLF	A	Q	G	V	S	V	D	K	G	V	E	T	D	S	S	K
H604-1/16	PQRETR↓GLF	A	Q	G	V	S	V	D	K	G	V	E	T	D	N	S	K
H1029-2/17	PQRETR↓GLF	A	Q	G	V	S	V	D	K	G	V	E	T	D	N	S	K
H422-7/16	PQRETR↓GLF	A	Q	G	V	A	V	D	K	G	V	E	T	D	S	S	R
A11-4/17	PQRETR↓GLF	A	Q	G	V	S	V	D	K	G	V	E	T	D	S	S	K
A14-2/17	PQRETR↓GLF	A	Q	G	V	S	V	D	K	G	V	E	T	D	S	S	K
H15-1/17	PQRETR↓GLF	A	Q	G	V	S	V	D	K	G	V	E	T	D	N	S	K
A09-1-2/17	PQRETR↓GLF	A	Q	G	V	A	V	D	K	G	V	E	T	D	N	S	K
A44-5/17	PQRETR↓GLF	A	Q	G	V	S	V	D	K	G	V	E	T	D	S	S	K
A33-5/17	PQRETR↓GLF	A	Q	G	V	A	V	D	K	G	V	E	T	D	N	N	K
H10-1/17	PQRETR↓GLF	A	Q	G	V	A	V	D	K	G	V	E	T	D	N	N	K
H55/17	PQRETR↓GLF	A	Q	G	V	S	V	D	K	G	V	E	T	D	N	S	K
H57-2/17	PQRETR↓GLF	A	Q	G	I	S	V	D	K	G	A	E	T	D	N	S	K
H1105-3/17	PQRETR↓GLF	A	Q	G	V	S	V	D	K	G	V	E	T	D	S	S	K
A32-3/17	PQRETR↓GLF	A	Q	G	V	A	V	D	K	G	V	E	T	D	N	S	K
A45-1/17	PQRETR↓GLF	A	Q	G	V	A	V	D	K	G	V	E	T	D	N	S	K
A46-1-4/17	PQRETR↓GLF	A	Q	G	V	A	V	D	K	G	V	E	T	D	N	S	K
A42-4/17	PQRETR↓GLF	A	Q	G	V	S	V	D	K	G	V	E	T	D	N	S	K
H51/17	PQRETR↓GLF	A	Q	G	V	S	V	D	K	G	V	E	T	N	N	S	K
A21-2/17	PQRETR↓GLF	A	Q	G	V	S	V	D	K	G	V	E	T	D	N	S	K
H33/17	PQRETR↓GLF	A	Q	G	V	A	V	D	K	G	V	E	T	D	S	S	K
H49/17	PQRETR↓GLF	A	Q	G	V	S	V	D	K	G	V	E	I	D	N	S	K
H112/17	PQRETR↓GLF	A	Q	G	V	A	V	D	K	G	V	E	T	D	N	S	K

^a^H5 numbering was used. HA, hemagglutinin; M, matrix; NS, nonstructural; PB, polymerase basic; PA, polymerase acidic.

### Phylogenetic analysis and genetic diversity of H5 LPAIVs

**Figure 1:**
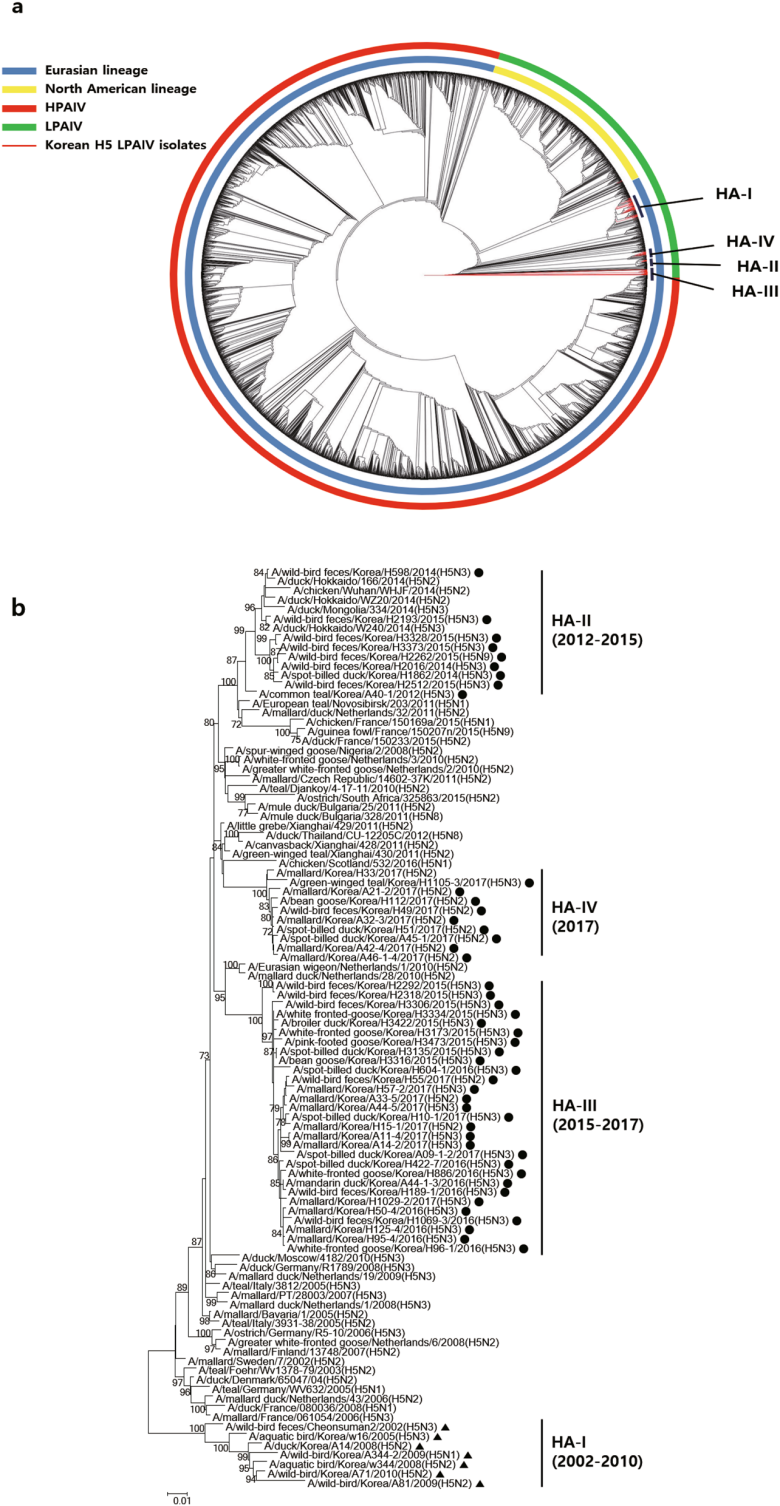
Phylogenetic analysis of the HA gene of H5 LPAIVs. (**A**) Maximum-likelihood phylogenetic tree of the HA genes of 48 H5 LPAIVs isolated in South Korea between 2010 and 2017 (red lines), along with all completed sequences (*n* = 5,283) of H5 genes of avian influenza viruses that were available in the using the NCBI Influenza Virus Database and GISAID. Evolutionary analyses were conducted in RAxML. (**B**) Maximum-likelihood phylogenetic tree of a subset of H5 genes belonging to the Eurasian lineage. The phylogenetic tree was generated in MEGA6. Bootstrap values (1,000 replicates) > 70% are displayed on the branch nodes. H5 LPAIVs isolated in South Korea from 2012 to 2017 are indicated by dots, and those isolated at other times are indicated by triangles. Sequences were acquired from GISAID and the NCBI Influenza Virus Database. HPAIV, highly pathogenic avian influenza virus; LPAIV, low pathogenic avian influenza virus.

Phylogenetic analyses showed that HA genes of all known H5 AIVs, including those identified in the current study, belonged to two geographically dependent lineages: Eurasian and North American (Fig. 1A). The HA genes of all H5 viruses identified in Korea since 2002 belonged to the Eurasian lineage and formed four distinct genetic subgroups (HA-I–IV) (Fig. 1B). Subgroup HA-I was composed of H5 LPAIVs identified up to 2010. The HA genes of the 48 H5 LPAIVs identified between 2012 and 2017 were distinct from those of HA-I, and were classified into three subgroups (HA-II–IV). The HA-II subgroup consisted of H5 LPAIVs detected in China, Japan, and South Korea between 2012 and 2015. The HA-III and HA-IV subgroups consisted of H5 LPAIVs identified in Korea in 2015–2017 (HA-III) and in 2017 only (HA-IV). For the neuraminidase gene, the phylogeny of N2 and N3 subtypes indicated that they belonged to the Eurasian lineage, with four distinct genetic subgroups for N2 and three for N3 (Supplementary Fig. 1A–B).

Phylogenetic analysis of internal genes of the 48 H5 LPAIVs identified between 2012 and 2017 in South Korea demonstrated a high level of heterogeneity. Multiple distinct genetic subgroups were identified for each gene segment. The PB2, PB1, and MP genes were of the Eurasian lineage, and clustered into 12, 9, and 4 distinct genetic groups, respectively (Supplementary Fig. 1C, D, G). Nearly all of the PA and NP sequences from H5 isolates in South Korea were of the Eurasian lineage, with the exception of the PA-XI and NP-VI subgroups, which belonged to the North American lineage (Supplementary Fig. 1E–F). The NS gene has previously been categorized into two distinct gene pools, classified as allele A and allele B^28^. All of the NS gene sequences belonged to the Eurasian lineage, with subgroups NS-I, NS-II, and NS-VII corresponding to allele A and subgroups NS-III–VI corresponding to allele B (Supplementary Fig. 1H).

**Figure 2:**
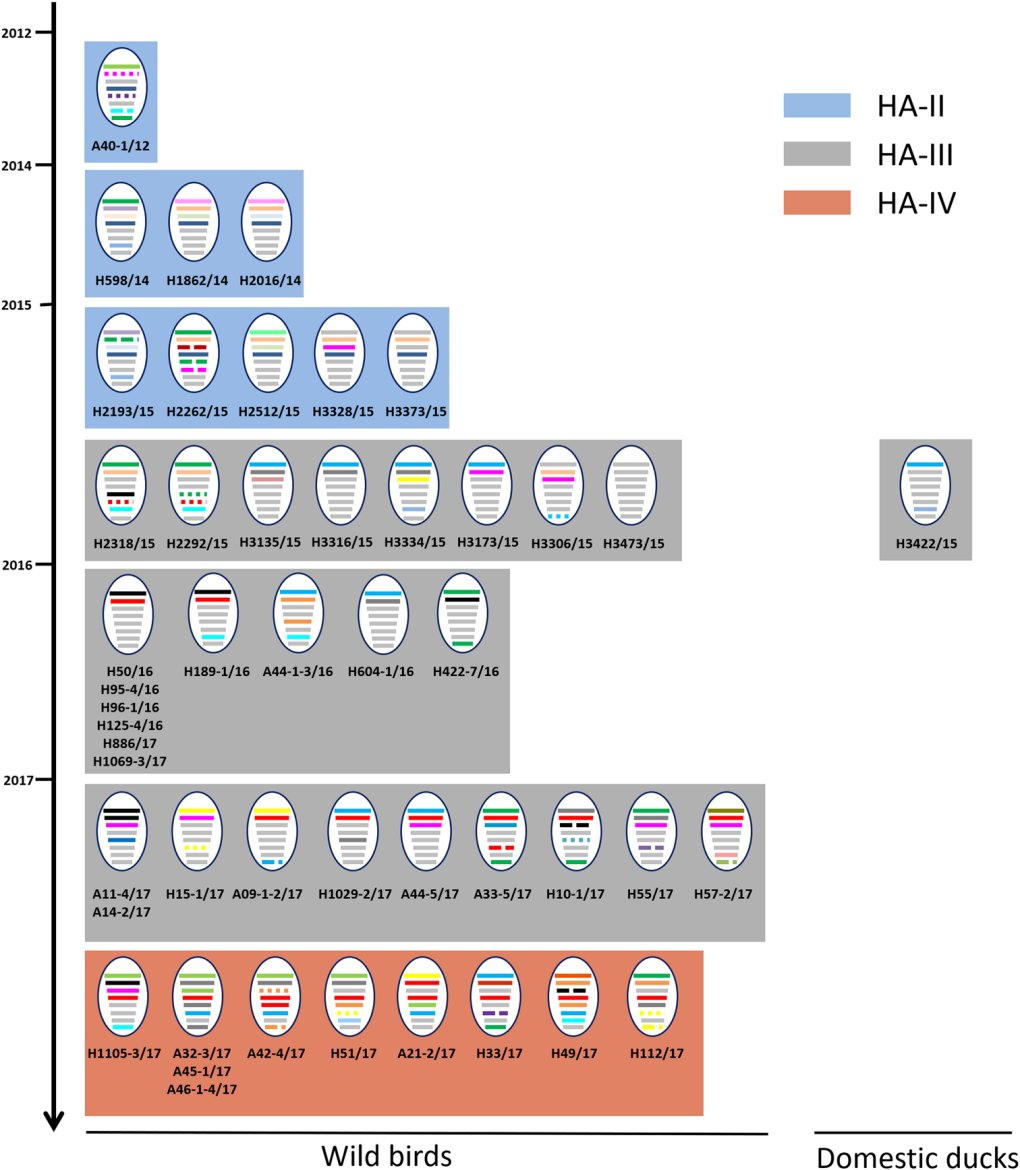
Genotypes of H5 LPAIVs isolated in South Korea from 2012 to 2017. The eight gene segments of avian influenza virus (from top to bottom, PB2, PB1, PA, HA, NP, NA, MP, and NS) are indicated by horizontal bars. The colors of the gene-segment bars denote different viral lineages. The timescale of virus isolation is indicated on the vertical scale. Labels below each genotype are identifiers for corresponding viruses characterized in this study.

The genotypes of H5 LPAIVs identified between 2012 and 2017 in South Korea were defined on the basis of gene-constellation analysis of each segment (with a requirement for > 97% nucleotide-sequence identity) and phylogenetic clustering (with a bootstrap value > 70). The 48 H5 LPAIVs identified from wild birds were classified into 39 distinct genotypes (Fig. 2). The HA-II subgroup was composed of nine distinct genotypes of viruses identified from the feces of spot-billed duck, bean goose, and unknown wild birds in several regions from December 2012 to December 2015, with 22 distinct genotypes of viruses identified from 2015 to 2017 in the HA-III subgroup, and eight distinct genotypes of viruses identified from April to December 2017 in the HA-IV subgroup.

In December 2015, one H5N3 virus (A/broiler duck/Korea/H3422/2015) was isolated from a domestic-duck farm in Jeollanam-do province in South Korea. The HA gene of this virus belonged to the HA-III subgroup. Moreover, the virus was genetically closely related to A/pink-footed goose/Korea/H3473/2015 for all gene segments except PB2 and MP, which were derived from A/white-fronted goose/Korea/H3334/2015-like virus.

### Replication and transmission of H5 LPAIVs in chickens

To investigate the replication and transmission capacities of representative strains of the three HA subgroups (HA-II–IV) in chickens, virus shedding via oropharyngeal and cloacal routes in both challenged and direct-contact groups was monitored for 2 weeks post-infection (p.i.) (Table 3). No mortality occurred in any of the chickens (data not shown). Nevertheless, in chickens challenged with A/spot-billed duck/H1862/2014(H5N3) of subgroup HA-II, virus shedding in oropharyngeal swabs began as early as day 1 p.i. and lasted until day 3 p.i. Moreover, the maximal viral loads in cloacal swabs (5.5 log_10_EID_50_/ml) were detected on day 7 p.i. On the basis of virus shedding and seroconversion, two of five chickens challenged with A/spot-billed duck/H1862/2014 (H5N3) virus were determined to have been infected.

**Table 3:** Replication and transmission of representative H5 low pathogenic avian influenza viruses in 3-week-old specific-pathogen-free chickens.

Isolate	Group	Sample	Numbers of infected birds (swab viral titers (log_10_EID_50_/ml)^a^)							Seroconversion (HI titer)^b^
			1 dpi	2 dpi	3 dpi	5 dpi	7 dpi	10 dpi	14 dpi	
A/spot-billed duck/Korea/H1862/2014(H5N3)	Challenged	OP	2/5 (1.5, 2.5)	1/5 (2.5)	1/5 (1.5)	0/5	0/5	0/5	0/5	2/5 (16, 16)
		CL	0/5	0/5	0/5	1/5 (3.5)	1/5 (5.5)	0/5	0/5	
	Direct contact	OP	0/3	0/3	0/3	0/3	0/3	0/3	0/3	0/3
		CL	0/3	0/3	0/3	0/3	0/3	0/3	0/3	
A/mallard/Korea/H95-4/2016(H5N3)	Challenged	OP	0/5	1/5 (3.5)	0/5	0/5	0/5	1/5 (1.5)	0/5	1/5 (16)
		CL	0/5	0/5	0/5	0/5	0/5	0/5	0/5	
	Direct contact	OP	0/3	0/3	0/3	0/3	0/3	0/3	0/3	0/3
		CL	0/3	0/3	0/3	0/3	0/3	0/3	0/3	
A/mallard/Korea/A32-3/2017(H5N2)	Challenged	OP	0/5	0/5	0/5	1/5 (3.5)	0/5	0/5	0/5	1/5 (16)
		CL	0/5	0/5	0/5	0/5	0/5	0/5	0/5	
	Direct contact	OP	0/3	0/3	0/3	0/3	0/3	0/3	0/3	0/3
		CL	0/3	0/3	0/3	0/3	0/3	0/3	0/3	

^a^Values shown are number of infected birds/number of inoculated birds in the challenge groups, and number of infected birds/number of naïve birds in the direct-contact groups. Values in parentheses are virus titers (log_10_EID_50_/ml). Mean viral titers from OP and CL swabs were calculated by the Reed and Muench method.

^b^Sera were collected from chickens at 14 dpi. Seroconversion was confirmed by HI assay. Values shown are number of birds showing seroconversion/number in group. Values in parentheses are HI titer.

CL, cloacal; dpi, days post-infection; EID_50_, 50% egg-infective dose; HI, hemagglutination inhibition; OP, oropharyngeal.

Viral shedding by the oropharyngeal route was detected in only one of five chickens that were challenged with A/mallard/Korea/H95-4/2016 (H5N3) of subgroup HA-III, and likewise in one of five chickens challenged with A/mallard/Korea/A32-3/2017 (H5N2) of subgroup HA-IV, with no viral shedding detected by the cloacal route in either case. Seroconversion was observed in one of five chickens challenged with each of these viruses. We did not detect virus in swabs from chickens in direct contact with any of the challenged birds, and neither was there any seroconversion in the direct-contact chickens.

To determine whether the representative LPAIVs replicated in chickens, we attempted to re-isolate virus from the tissues of birds euthanized on day 4 p.i. None of the three representative viruses demonstrated detectable replication in the examined tissues (data not shown). No macroscopic/microscopic lesion or virus was detected in any of the examined organs of infected chickens.

### Replication and pathogenicity of H5 LPAIVs in mice

**Figure 3:**
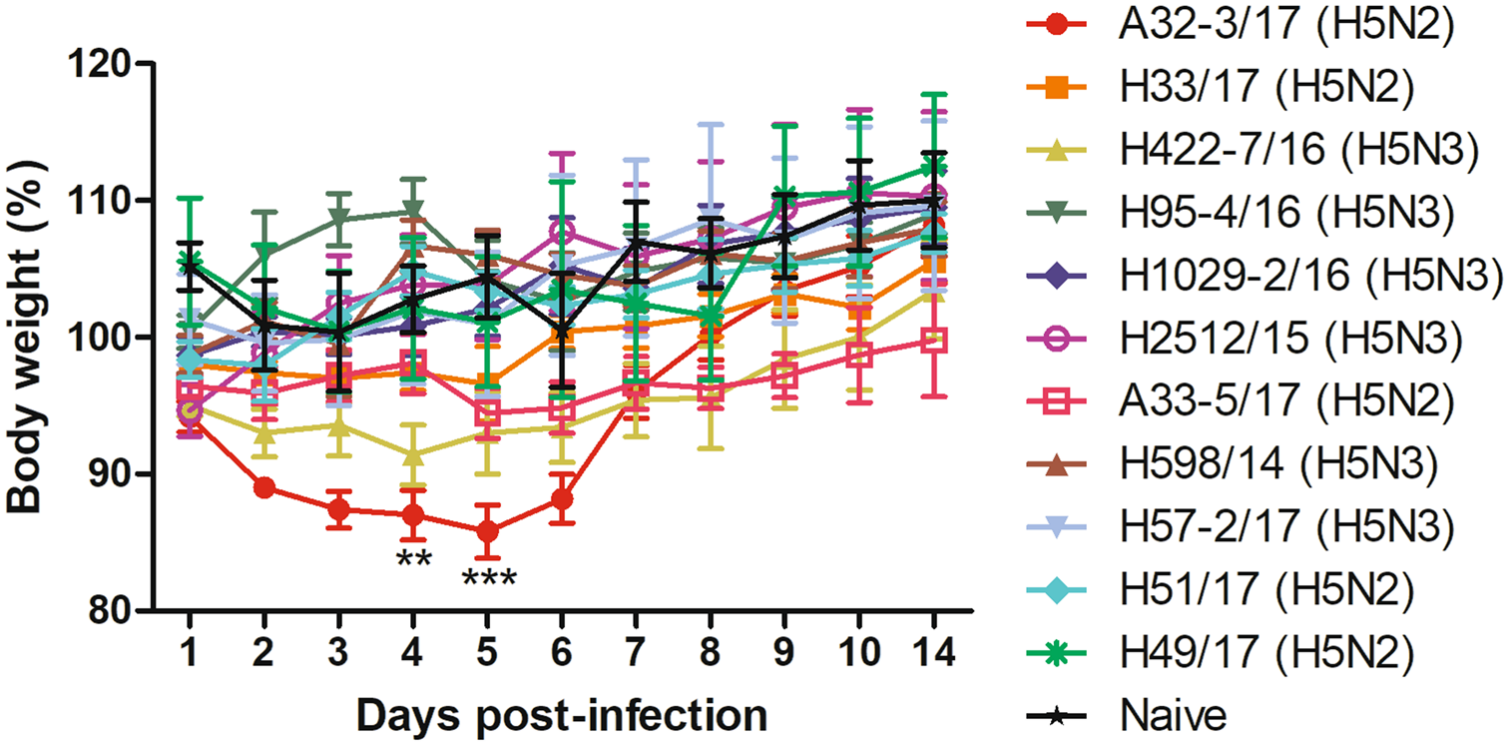
Pathogenicity of representative strains of H5 LPAIVs in mice, indicated by changes in body weight. BALB/c mice (*n* = 5 per group) were challenged with 10^6^ 50% egg-infective doses per animal of each virus. Changes in body weight were observed for 14 days. No mortality occurred. Data points indicate means, and error bars indicate SEM. Data were analyzed by two-way ANOVA with Bonferroni’s post-test. Asterisks indicate statistical significance of the difference in body weight between naïve mice and mice infected with A32-3/17 (H5N2) virus (**p < 0.01; ***p < 0.001).

To evaluate replication by the H5 LPAIVs, groups of mice were intranasally inoculated with 50 µl per animal of 2 × 10^7^ 50% egg-infective doses (EID_50_)/ml of virus. Representative isolates (*n* = 11) were selected on the basis of the presence of known mammalian-adaptation markers (Table 2). All challenged mice survived until the final assessment on day 14 p.i. (Fig. 3) and showed no obvious signs of disease, with the exception of mice infected with A/mallard/Korea/A32-3/2017 (H5N2) virus, which had an initial period of weight loss, resulting in significantly lower body weight than naïve mice on day 4 p.i. (*p* < 0.01) and day 5 p.i. (*p* < 0.001).

On day 3 p.i., 10 of the 11 representative H5 LPAIVs (but not A/wild bird feces/Korea/H49/2017(H5N2)) were detected in the lungs of challenged mice, with mean titers of 2.5–6.3 log_10_EID_50_/g (Table 4). Notably, we isolated A/mallard/Korea/A32-3/2017 at 1.75 log_10_EID_50_/g in the brain of one of three challenged mice, and likewise we isolated A/wild bird feces/Korea/H33/2017 at 1.5 log_10_EID_50_/g in the brain of another mouse, and A/mallard/Korea/H1029-2/2016 at 2.5 log_10_EID_50_/g in the kidney of a third mouse.

**Table 4:** Virus titers in tissues from 6-week-old female BALB/c mice challenged with representative H5 isolates.

Isolate	Tissue		
	Lung	Kidney	Brain
A/mallard/Korea/A32-3/2017(H5N2)	3/3 (5.41 ± 0.63)	0/3	1/3 (1.75)
A/wild bird feces/Korea/H33/2017(H5N2)	3/3 (4.16 ± 0.39)	0/3	1/3 (1.5)
A/spot-billed duck/Korea/H422-7/2016(H5N3)	3/3 (4.91 ± 1.02)	0/3	0/3
A/mallard/Korea/H95-4/2016(H5N3)	3/3 (4.83 ± 1.15)	0/3	0/3
A/mallard/Korea/H1029-2/2017(H5N3)	4/4 (6.25 ± 0.5)	1/4 (2.5)	0/4
A/wild bird feces/Korea/H2512/2015(H5N3)	2/3 (2.75 ± 0.35)	0/3	0/3
A/mallard/Korea/A33-5/2017(H5N2)	3/4 (3.83 ± 1.15)	0/4	0/4
A/wild bird feces/Korea/H598/2014(H5N3)	1/4 (5.5)	0/4	0/4
A/wild bird feces/Korea/H57-2/2017(H5N3)	2/4 (2.5 ± 0.71)	0/4	0/4
A/spot-billed duck/Korea/H51/2017(H5N2)	3/3 (3.66 ± 0.29)	0/3	0/3
A/wild bird feces/Korea/H49/2017(H5N2)	0/3	0/3	0/3

Values shown are number of mice with virus in the tissue/number of inoculated mice. Values in parentheses are virus titers (log_10_EID_50_/g). For multiple infected tissues, the virus titer is the mean ± standard deviation of the samples.

EID_50_, 50% egg-infective dose.

## Discussion

Since 2008, nationwide surveillance program for avian influenza has been developed in South Korea. Our results have now shown that the annual prevalence of AIVs in samples from wild-bird habitats in South Korea was 0.2–1.5% from 2010 to 2017, which is consistent with values of 0.4–1.2% for major wild-bird habitats in South Korea from 2003 to 2008^29^. In Japan, the results of intensive surveillance between 2010 and 2014 demonstrated annual AIV prevalence of 1.9–4.0%^30^. Wild waterfowl can harbor diverse AIVs, and these viruses may be transferred to domestic poultry along the flyways. Therefore, overwintering and stopover sites on migration routes should be monitored continuously for AIVs through systematic surveillance.

The ability of the H5 and H7 subtypes of LPAIVs to mutate to HPAIVs in poultry is the main reason for their stringent control. Previously, two H5N2 LPAIVs were isolated from duck farms in South Korea through national active surveillance for AIV in 2008, and were found to be genetically related to LPAIVs circulating in Eurasia^31^. Moreover, 10 H7 LPAIVs were also detected in live-bird markets and domestic-duck farms between 2008 and 2011^32^. Even when these viruses were characterized as low pathogenic forms, rapid eradication was accomplished through depopulation of infected flocks, quarantine, and inspection of epidemiologically related farms. Indemnities were paid for destruction of poultry. In the period from 2011 to 2017, we identified no H7 LPAIV and only one H5N3 LPAIV (A/broiler duck/Korea/H3422/2015) by surveillance of terrestrial poultry such as chicken farms, duck farms, and live-poultry markets. The H5 LPAIV was isolated from a duck farm located in southwestern South Korea (a region that is characterized by high wild-bird and domestic-duck density) during the winter season, which is associated with the migration of overwintering wild waterfowl into the region. Domestic ducks are known to be intermediate hosts between migratory waterfowls and terrestrial poultry^33,34,35^. Because small-holder duck farms belong to sector 3 production systems, with low-to-minimal biosecurity, domestic ducks may serve as asymptomatic carriers of IAV to other species through the live-poultry trading network. Eradication protocols presumably eliminated the H5 LPAIV from domestic ducks, preventing the virus from entering live-poultry markets and chicken farms, and thereby eliminating the opportunities for the virus to evolve to a highly pathogenic form. To prevent the evolution of LPAIVs to HPAIVs, systematic surveillance for H5 and H7 LPAIVs should be adopted in combination with an immediate-eradication strategy.

In the study described herein, we genetically characterized 48 H5 LPAIVs isolated in South Korea from 2010 to 2017. Our results showed that HA-I-subgroup H5 viruses detected from 2002 to 2010 were replaced by HA-II-subgroup viruses from 2012 onwards. Although the mean annual proportion of H5 LPAIVs among AIV isolations was 5.3% between 2010 and 2017, detection rates were particularly high for HA-III viruses in 2015 (13.1%) and HA-IV viruses in 2017 (14.4%). Previously, we reported that H7 LPAIVs isolated from wild-bird habitats in the winter of 2016–2017 constituted ≥ 11 distinct genotypes, with unusually high prevalence (43.6%) among AIV isolates^36^. Similarly, the H5 LPAIVs that we have now described comprised ≥ 35 distinct genotypes from 2015 to 2017, representing a high level of genetic diversity. Long-term surveillance in North America has identified a correlation between peaks of prevalence in wild waterfowl and the emergence of H7 AIVs in domestic poultry^37^. In the 2017 HPAI outbreaks in the southeastern USA, H7N9 mutation from LPAIV to HPAIV occurred in poultry, and whole-genome sequencing and comparative genetic analyses of all available sequences of the North American wild-bird H7N9 lineage identified a wild-bird-origin precursor virus with the identical genome constellation^38^. Therefore, recent population expansions and subsequent increases in genetic diversity of the H5 LPAIVs harbored by overwintering wild waterfowl highlight the need for enhanced active-surveillance systems for wild-bird habitats to monitor the spillover of AIVs into terrestrial poultry.

Between 1959 and 2016, 15 outbreaks of H5 HPAI were reported worldwide (excluding Gs/Gd-lineage H5 HPAI)^10,39–41^. In five of these outbreaks, an LPAIV precursor was detected among chickens, turkeys, or ostriches prior to the HPAIV emergence. Therefore, the detection and spread of H5 LPAIVs in gallinaceous poultry or ostriches are considered to be signs of HPAIV emergence. It has been reported that Eurasian H5 LPAIVs do not replicate and transmit in chickens, whereas some strains of H7 LPAIVs can be recovered at high titers in experimentally infected chickens^31,32,36,42^. Consistently, we found that replication of three representative strains of the H5 LPAIV HA-I–III subgroups in chickens was restricted, and virus was not detected in tissues at day 4 p.i., indicating that these H5 viruses were not well adapted for infection of gallinaceous poultry. Nonetheless, with each virus, we detected some shedding in challenged birds. In previous studies, virus was not detected in oropharyngeal or cloacal swabs from chickens challenged with H5 LPAIVs, suggesting that recently isolated Eurasian H5 LPAIVs are better adapted to chickens than viruses isolated before 2010. Interestingly, an H5N1 LPAIV (A/chicken/Scotland/532/2016(H5N1)) that was detected in broiler breeder chickens in Scotland in January 2016 was found to have rapid within-farm spread^43^. Although this virus had the HA cleavage-site motif of an LPAIV belonging to the Eurasian H5 lineage, histopathology and immunohistochemistry revealed that viral antigen was observed in the endothelial cells of the blood vessels of brain and viscera of infected chickens, suggesting systemic infection. These observations indicate that systematic surveillance of the breeding and overwintering sites of Eurasia, and timely evaluation of the pathogenic potential of H5 or H7 LPAIVs are essential to provide early warnings of possible adaptations to terrestrial poultry or evolution into highly pathogenic forms.

In mice that were challenged with H5 LPAIVs, we found that 10 of the 11 viruses used infected and replicated in mice without prior adaptation, and some caused a degree of weight loss, which is consistent with previous findings for H5 LPAIVs^44^. Although these H5 LPAIVs did not contain the mammalian-adaptive substitution E627K, they replicated efficiently in murine lungs, suggesting the presence of other variations in the polymerase complex that are responsible for host adaptation. We identified a number of variations in the polymerase subunits of H5 LPAIVs, including PB2 D701N, PB1 R198K, PB1-F2 N66S, and PA S409N and K615R. Moreover, we found that the representative viruses each had some or all of the mutations in the PB2 gene that correspond to the L89V, G309D, T339K, R477G, I495V, and A676T substitutions, which can compensate for a lack of the E627K substitution^22^. In publicly available sequences, we found that a combination of six mutations and 627E in PB2 was identified in 58 of 340 AIVs responsible for human infections, including H5N1, H7N7 and H9N2, indicating that the PB2 627 K was not a sole determinant factor of adaptation of AIVs in mammals. Moreover, 67.5% (13,074/19,338) of IAVs isolated from avian hosts possess a combination of these mutations in PB2. It has been reported that numerous LPAIVs isolated from wild birds can replicate in the BALB/c mouse model without prior adaptation^45–48^. In addition to standard laboratory mice model, previous experimental infection study has shown that several subtypes of LPAIVs were able to infect and efficiently replicate in wild house mice model without adaptation^49^. Therefore, further characterization is now needed of the molecular determinants of the replication of LPAIVs, and of how these adaptation markers may interact in a synergistic manner in mammals.

Wild waterfowl in Eurasia maintain a large genetic pool of AIVs, which contributes to the emergence of novel strains that can establish infections in new hosts, such as domestic poultry and mammals. The recent unusually high frequency of H5 LPAIV detection and the associated genetic diversity emphasize the need for continued surveillance in both wild birds and poultry in Eurasia, along with risk assessment by the use of molecular analysis and in vivo models.

## Materials and methods

### Ethics statement

Experiments in animals were evaluated by the Institutional Animal Care and Use Committee of the Animal and Plant Quarantine Agency (APQA) in South Korea, and gained approval (2017–383 and 2018–418). All procedures were performed in accordance with the relevant guidelines and regulations and carried out in a biosafety level 2 facility at the APQA.

### Sample collection and virus isolation

A total of 85,588 samples from wild-bird habitats and 753,535 samples from domestic-duck farms were collected according to the national surveillance program in South Korea between 2010 and 2017. Sample collection was conducted by the Livestock Health Control Association or by regional veterinary offices. In major migratory habitats, oropharyngeal and cloacal swabs were collected from captured birds. Carcasses of birds and fecal samples were also collected. In domestic-duck farms, fecal samples, carcasses, and oropharyngeal and cloacal swabs were also collected.

Swabs, fecal samples, and tissue homogenates from dead birds were examined by virus isolation in 9–11-day-old specific-pathogen-free (SPF) embryonated eggs. The presence of IAV in the allantoic fluid was determined by hemagglutination assay using chicken erythrocytes after incubation for 4 days at 37 °C. AIV-positive fecal samples were analyzed by a DNA barcoding system for host-species identification, as previously described^50^.

### Genome-sequence generation and phylogenetic analysis

AIV genomes were amplified from extracted RNA using universal and gene-specific primers with an Omniscript reverse-transcription kit (QIAGEN, Germantown, MD, USA) and an Ex Taq polymerase (TAKARA, Kusatsu, Japan) on a Sanger sequencing platform^51^.

To perform systematic analysis, all available sequence information was retrieved in May 2018 for isolates collected in Asia and North America over the period of this study, using the National Center for Biotechnology Information (NCBI) Influenza Virus Database (https://www.ncbi.nlm.nih.gov/genomes/FLU/Database/nph-select.cgi) and the Global Initiative on Sharing All Influenza Data (GISAID; https://www.gisaid.org). Multiple-sequence alignments were generated with the MAFFT tool^52^. Phylogenetic trees for gene segments were constructed with a maximum-likelihood method by running RAxML v8.2.10. A maximum-likelihood phylogenetic tree of a subset of H5 genes was generated in MEGA (version 6). A general time reversible nucleotide substitution model with gamma-distributed rate variation among sites was applied throughout the analysis. To analyze for amino acid substitutions, full-length protein sequences of PB2 of IAVs isolated from human and avian were collected from the database on February 2020 and aligned with the corresponding sequences of the LPAI isolates.

### Genotype analyses

To describe the genetic diversity of IAVs detected in South Korea, clusters were defined for each gene-segment-specific phylogeny by use of the following criteria: sharing a phylogenetic cluster with a minimum bootstrap value of 70, and sequences having > 97% nucleotide-sequence identities. Each genotype was the combination of the cluster assignment of eight gene segments.

### Experimental-animal infection

#### Chickens

SPF white leghorn chickens (3 weeks old, *n* = 8 per group for each virus) were inoculated intranasally with 100 µl per animal of 10^7^ EID_50_/ml virus. The viral challenge dose in the inoculum was confirmed by immediate back titration in SPF eggs. At 8 h p.i., three naïve chickens were housed together with the inoculated chickens. The chickens were monitored daily for 14 days and checked for morbidity and mortality. Oropharyngeal and cloacal swabs were collected on days 3, 5, 7, 10, and 14 p.i. For virus replication in organs, groups of chickens (*n* = 3 per group) were euthanized on day 4 p.i. Organs (trachea, cecal tonsil, lung, spleen, kidney, brain, and pancreas) were collected for virus titration by use of egg-infection assays. On day 14 p.i., sera collected from chickens in each group were tested by hemagglutination-inhibition assays, according to International Epizootic Office (OIE) recommendations.

#### Mice

Female BALB/c mice (6 weeks old, *n* = 5 per group for each virus; Orient-Bio, South Korea) were intranasally inoculated with 50 µl per animal of 2 × 10^7^ EID_50_/ml virus, while under light anesthesia induced by Avertin (Sigma-Aldrich, Milan, Italy). Mice were observed daily for 14 days and checked for changes in body weight, and for mortality. Mice were euthanized if they lost > 25% of their initial body weight. On day 3 p.i., three mice from each group were euthanized. Organs (lung, liver, spleen, kidney, and brain) were collected for virus titration by use of egg-infection assays. On day 14 p.i., sera collected from mice in each group were also tested by hemagglutination-inhibition assays.

## Supplementary information

Supplementary file1
