# Supplementary material for: Genetic characteristics and pathogenesis of H5 low pathogenic avian influenza viruses from wild birds and domestic ducks in South Korea
Source: Sci Rep. 2020 Jul 22;10:12151. doi: 10.1038/s41598-020-68720-w (PMC7376034; doi:10.1038/s41598-020-68720-w)
Supplement: Supplementary file 1 — Supplementary file1 [file 41598_2020_68720_MOESM1_ESM.pdf]

Supplementary information

Genetic characteristics and pathogenesis of H5 low pathogenic avian influenza viruses from wild birds and domestic ducks in South Korea

Yu-Na Lee[1], Dong-Hun Lee[2], Sun-Ha Cheon[1], Yu-Ri Park[1], Yoon-Gi Baek[1], Young-Jae Si[1], Soo-Jeong Kye[1], Eun-Kyoung Lee[1], Gyeong-Beom Heo[1], You-Chan Bae[3], Myoung-Heon Lee[1], and Youn-Jeong Lee[1,*]

Supplementary Table 1. H5 low pathogenic avian influenza viruses isolated in South Korea between 2012 and 2017.

Genotype	Virus name	Subtype	Collection date	Region	Samples	GISAID Isolate ID
HA-II	A/common teal/Korea/A40-1/2012	H5N3	2012-12-18	JJ	Feces	EPI_ISL_243044
	A/wild bird feces/Korea/H598/2014	H5N3	2014-02-18	Seoul	Feces	EPI_ISL_243045
	A/spot-billed duck/Korea/H1862/2014	H5N3	2014-11-14	GG	Swab	EPI_ISL_369299
	A/wild bird feces/Korea/H2016/2014	H5N3	2014-12-24	GN	Feces	EPI_ISL_243047
	A/wild bird feces/Korea/H2193/2015	H5N3	2015-01-07	CN	Feces	EPI_ISL_369292
	A/wild bird feces/Korea/H2262/2015	H5N9	2015-01-15	Busan	Feces	EPI_ISL_369293
	A/wild bird feces/Korea/H2512/2015	H5N3	2015-03-09	JJ	Feces	EPI_ISL_369294
	A/wild bird feces/Korea/H3328/2015	H5N3	2015-11-19	GG	Feces	EPI_ISL_369308
	A/bean goose/Korea/H3373/2015	H5N3	2015-12-07	Seoul	Feces	EPI_ISL_369332
HA-III	A/wild bird feces/Korea/H2292/2015	H5N3	2015-01-22	GG	Feces	EPI_ISL_377143
	A/wild bird feces/Korea/H2318/2015	H5N3	2015-01-21	GG	Feces	EPI_ISL_369295
	A/spot-billed duck/Korea/H3135/2015	H5N3	2015-11-02	CN	Feces	EPI_ISL_369300
	A/white-fronted goose/Korea/H3173/2015	H5N3	2015-10-29	GW	Feces	EPI_ISL_369301
	A/wild bird feces/Korea/H3306/2015	H5N3	2015-11-09	GG	Feces	EPI_ISL_369296
	A/bean goose/Korea/H3316/2015	H5N3	2015-11-16	GG	Feces	EPI_ISL_369309
	A/white-fronted goose/Korea/H3334/2015	H5N3	2015-11-23	GG	Feces	EPI_ISL_369302
	A/broiler duck/Korea/H3422/2015	H5N3	2015-12-17	JN	Carcasses	EPI_ISL_369297
	A/pink-footed goose/Korea/H3473/2015	H5N3	2015-12-21	CN	Feces	EPI_ISL_369303
	A/mandarin duck/Korea/A44-1-3/2016	H5N3	2016-11-04	JN	Feces	EPI_ISL_369310
	A/mallard/Korea/H50-4/2016	H5N3	2016-11-22	CN	Feces	EPI_ISL_369304
	A/mallard/Korea/H95-4/2016	H5N3	2016-11-29	JB	Feces	EPI_ISL_369305
	A/white-fronted goose/Korea/H96-1/2016	H5N3	2016-11-29	GG	Feces	EPI_ISL_369306
	A/mallard/Korea/H125-4/2016	H5N3	2016-12-01	JB	Feces	EPI_ISL_369307
	A/wild bird feces/Korea/H189-1/2016	H5N3	2016-12-01	GN	Feces	EPI_ISL_369298
	A/spot-billed duck/Korea/H422-7/2016	H5N3	2016-12-14	GB	Feces	EPI_ISL_369311
	A/spot-billed duck/Korea/H604-1/2016	H5N3	2016-12-27	GN	Feces	EPI_ISL_369312
	A/white-fronted goose/Korea/H886/2017	H5N3	2017-01-16	JB	Feces	EPI_ISL_369313
	A/mallard/Korea/H1029-2/2017	H5N3	2017-03-13	GG	Feces	EPI_ISL_369314

	A/wild bird/Korea/H1069-3/2017	H5N3	2017-03-20	JJ	Feces	EPI_ISL_369315
	A/spot-billed duck/Korea/A09-1-2/2017	H5N3	2017-10-10	GG	Feces	EPI_ISL_369333
	A/mallard/Korea/A11-4/2017	H5N3	2017-10-10	Seoul	Feces	EPI_ISL_369316
	A/mallard/Korea/A14-2/2017	H5N3	2017-10-10	Seoul	Feces	EPI_ISL_369317
	A/mallard/Korea/A33-5/2017	H5N2	2017-11-06	JJ	Feces	EPI_ISL_369318
	A/mallard/Korea/A44-5/2017	H5N3	2017-11-15	JB	Feces	EPI_ISL_369319
	A/spot-billed duck/Korea/H10-1/2017	H5N3	2017-11-20	GG	Feces	EPI_ISL_369334
	A/mallard/Korea/H15-1/2017	H5N2	2017-11-22	JB	Feces	EPI_ISL_369320
	A/wild bird/Korea/H55/2017	H5N2	2017-12-13	GG	Feces	EPI_ISL_369321
	A/wild bird/Korea/H57-2/2017	H5N3	2017-12-18	JB	Feces	EPI_ISL_369322
HA-IV	A/green-winged teal/Korea/H1105-3/2017	H5N3	2017-04-20	CN	Feces	EPI_ISL_369323
	A/mallard/Korea/A21-2/2017	H5N2	2017-10-26	CB	Feces	EPI_ISL_369324
	A/mallard/Korea/A32-3/2017	H5N2	2017-11-06	GG	Feces	EPI_ISL_369325
	A/mallard/Korea/A42-4/2017	H5N2	2017-11-13	CN	Feces	EPI_ISL_369326
	A/spot-billed duck/Korea/A45-1/2017	H5N2	2017-11-15	CN	Feces	EPI_ISL_369327
	A/mallard/Korea/A46-1-4/2017	H5N2	2017-11-15	GG	Feces	EPI_ISL_369328
	A/wild bird feces/Korea/H33/2017	H5N2	2017-12-06	JN	Feces	EPI_ISL_369329
	A/wild bird feces/Korea/H49/2017	H5N2	2017-12-06	CN	Feces	EPI_ISL_369335
	A/spot-billed duck/Korea/H51/2017	H5N2	2017-12-11	CN	Feces	EPI_ISL_369330
	A/bean goose/Korea/H112/2017	H5N2	2017-12-27	JN	Feces	EPI_ISL_369331

GISAID, Global Initiative on Sharing All Influenza Data; GG, Gyeonggi-do; GN, Gyeongsangnam-do; CN, Chungcheongnam-do; JJ, Jeju-do; GW, Gangwon-do; JN, Jeollanam-do; JB, Jeollabuk-do; GB, Gyeongsangbuk-do; CB, Chungcheongbuk-do.

Supplementary Figure 1. Phylogenetic analysis of individual genes of H5 LPAIVs.

Maximum-likelihood phylogenetic trees of (A) N2, (B) N3, (C) PB2, (D) PB1, (E) PA, (F) NP, (G) MP, and (H) NS genes of 48 H5 LPAIVs isolated in South Korea between 2010 and 2017 (red lines), along with all completed sequences (black lines) of N2 (*n* = 4,778), N3 (*n* = 1,576), PB2 (*n* = 10,500), PB1 (*n* = 10,627), PA (*n* = 9,492), NP (*n* = 9,959), MP (*n* = 9,963), and NS (*n* = 9,426) genes of avian influenza viruses that were available in GISAID and the NCBI Influenza Virus Database. Evolutionary analyses were conducted in RAxML.

LPAIV, low pathogenic avian influenza virus.

a N2

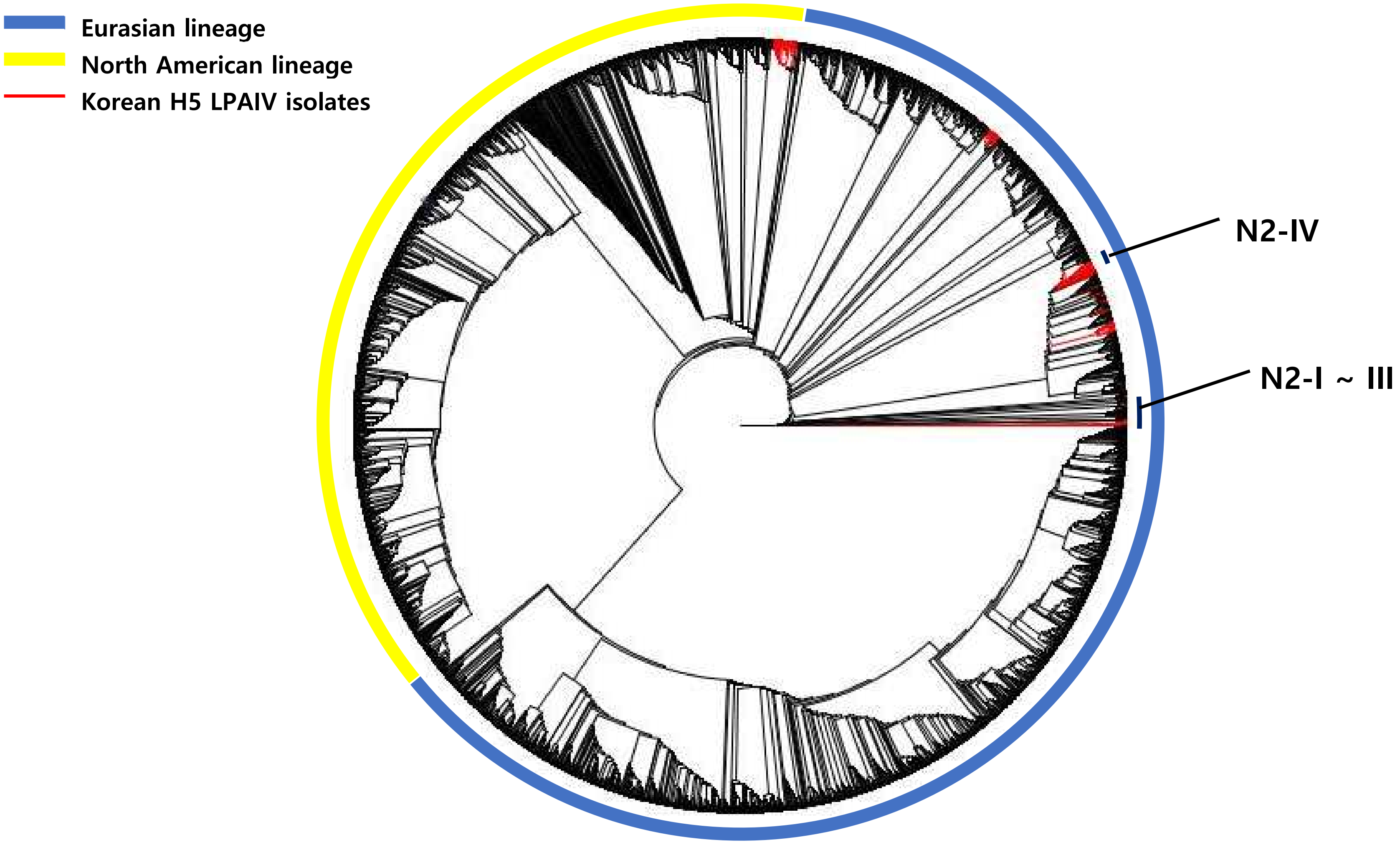
Eurasian lineage
North American lineage
Korean H5 LPAIV isolates
N2-IV
N2-I ~ III

b N3

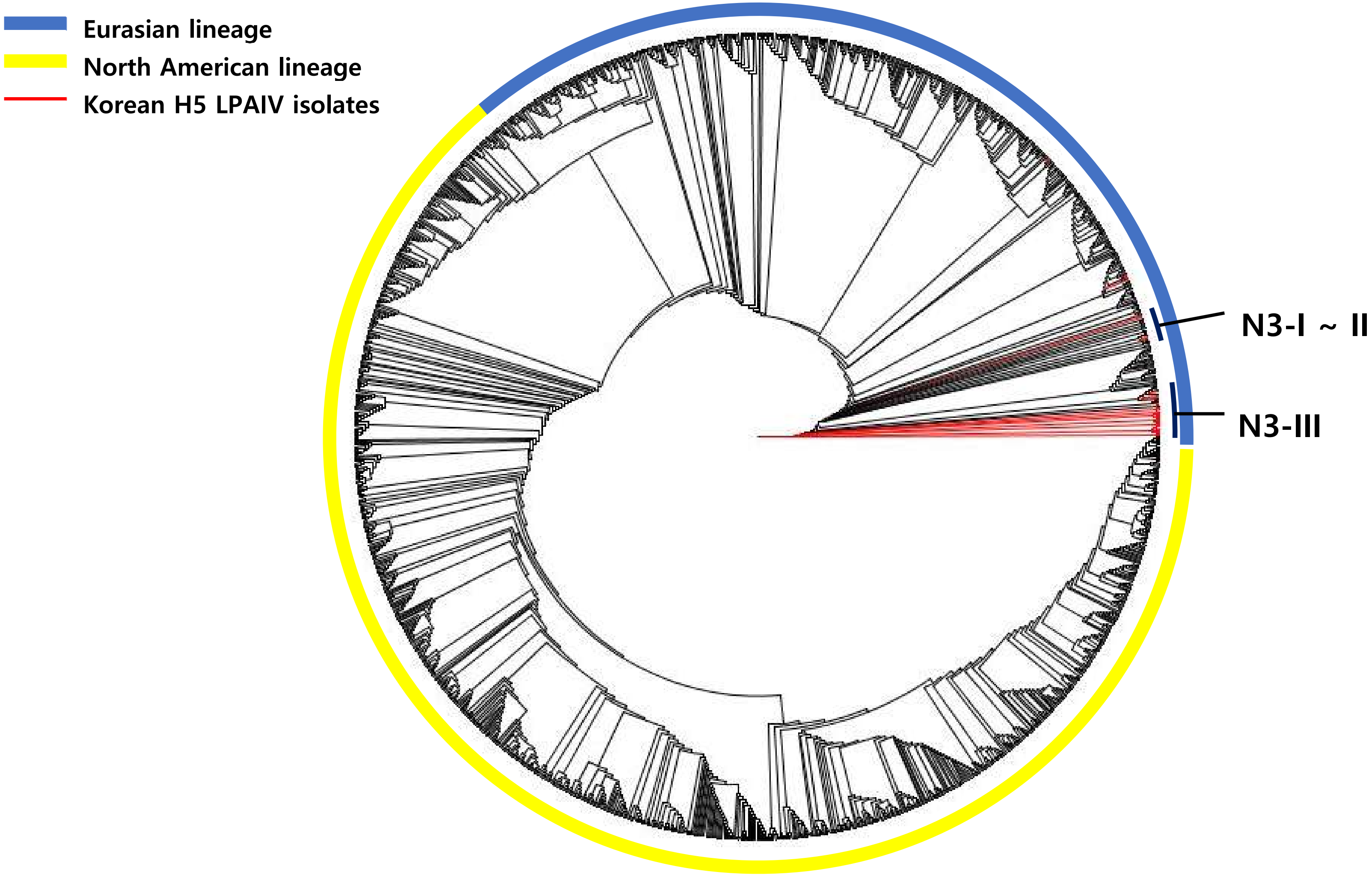

Eurasian lineage
North American lineage
Korean H5 LPAIV isolates
N3-I ~ II
N3-III

c PB2

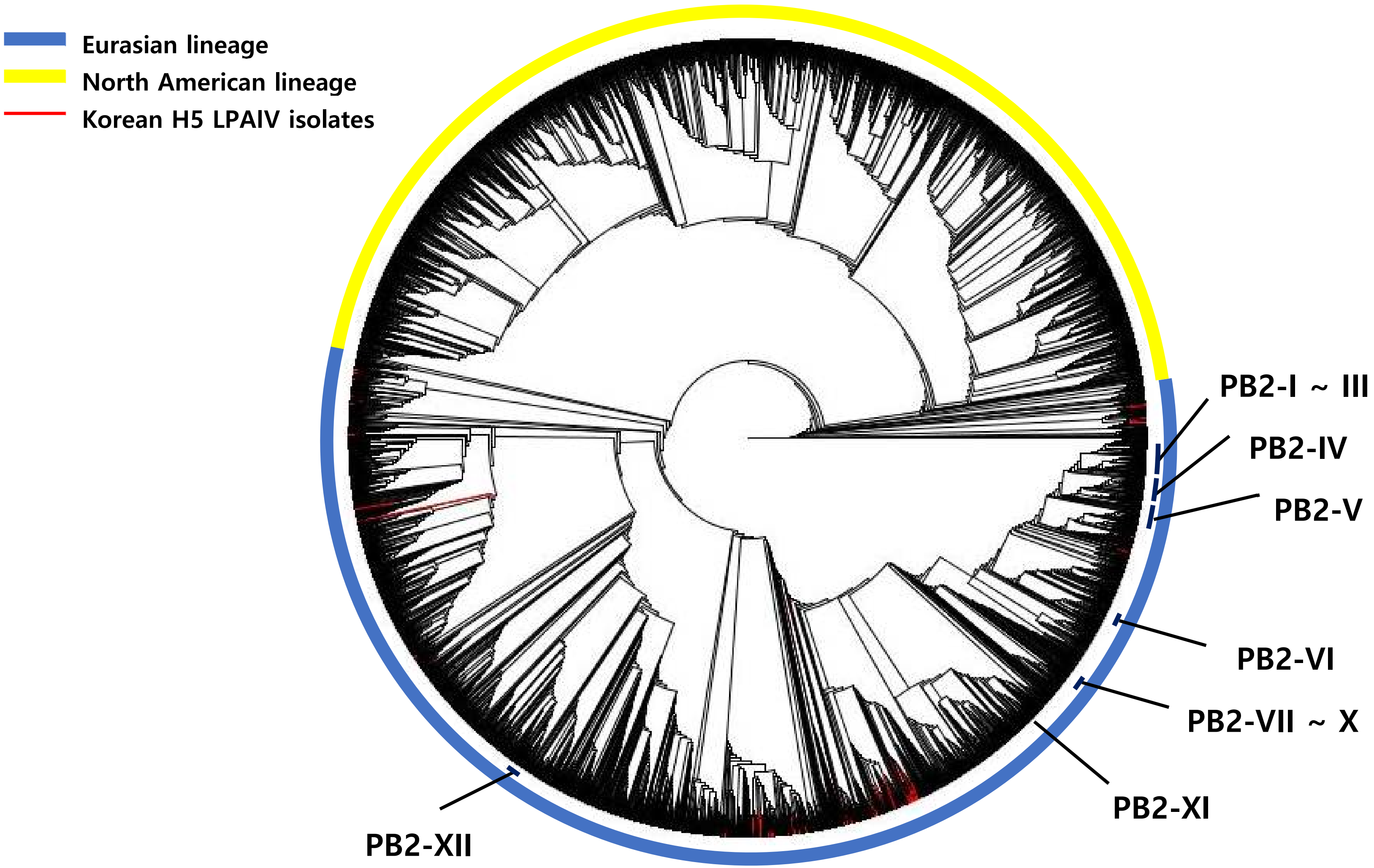

Eurasian lineage
North American lineage
Korean H5 LPAIV isolates
PB2-I ~ III
PB2-IV
PB2-V
PB2-VI
PB2-VII ~ X
PB2-XI
PB2-XII

d PB1

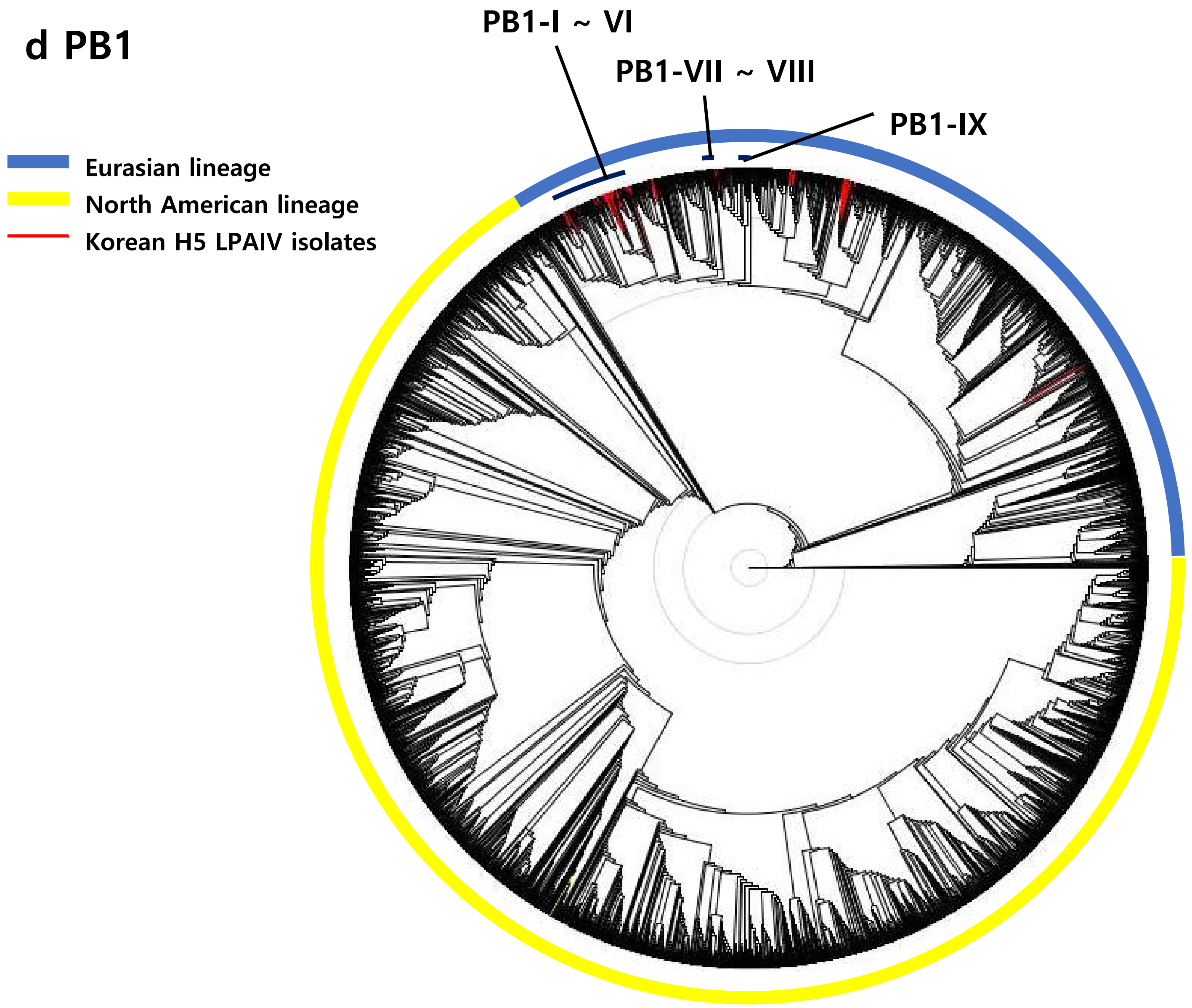
PB1-I ~ VI
PB1-VII ~ VIII
PB1-IX
Eurasian lineage
North American lineage
Korean H5 LPAIV isolates

e PA

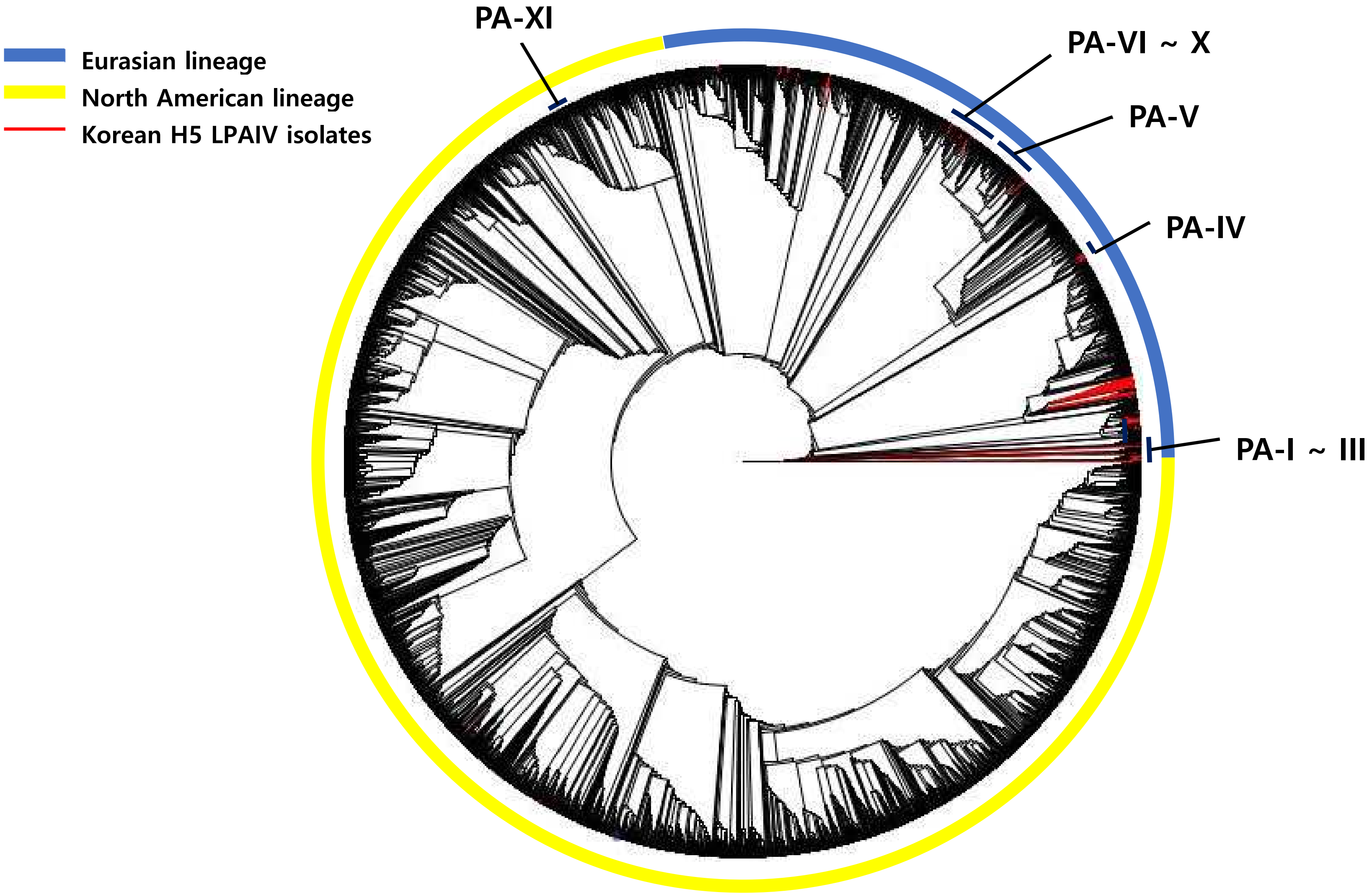
Eurasian lineage
North American lineage
Korean H5 LPAIV isolates
PA-XI
PA-VI ~ X
PA-V
PA-IV
PA-I ~ III

f NP

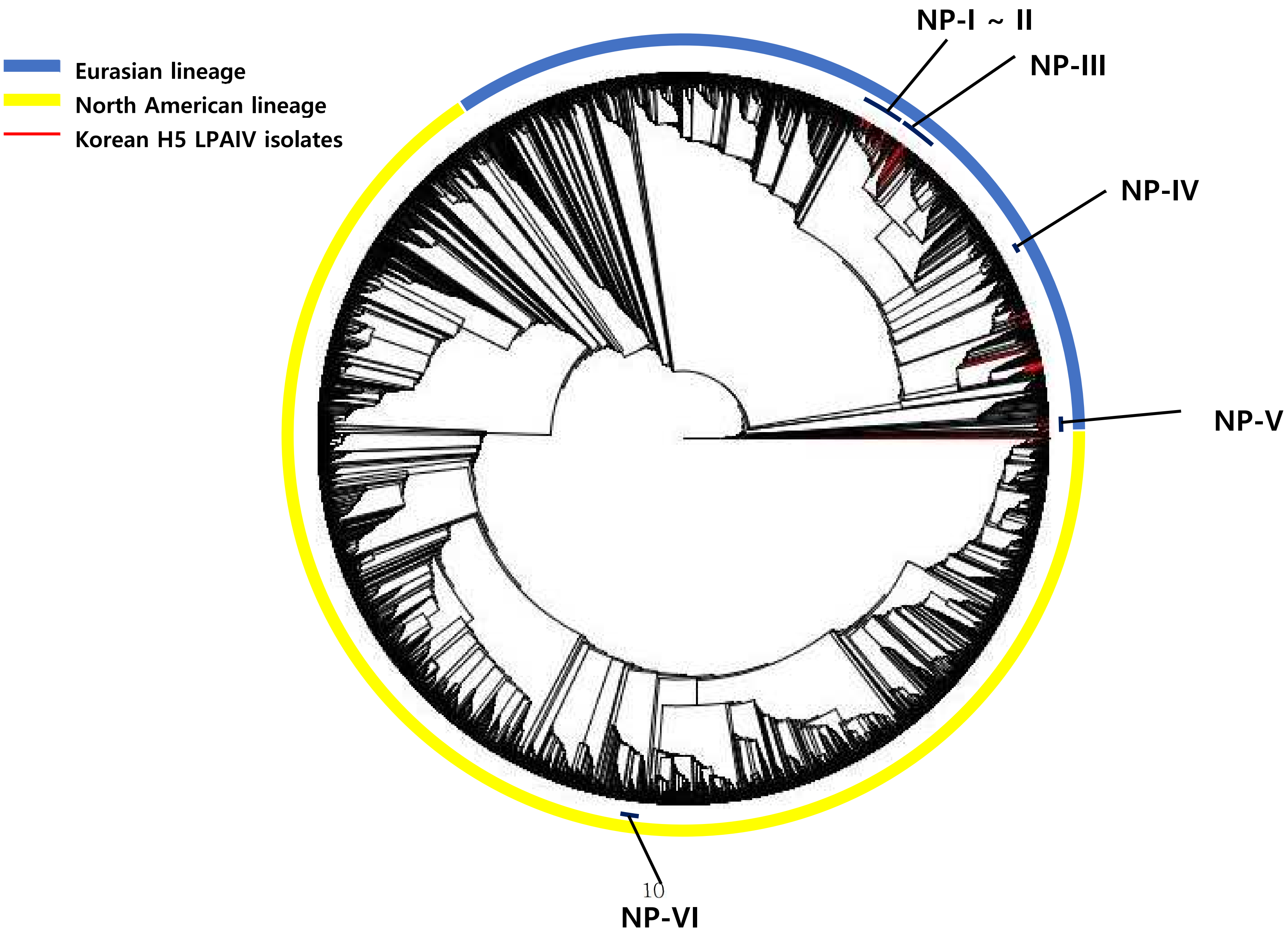
Eurasian lineage
North American lineage
Korean H5 LPAIV isolates
NP-I ~ II
NP-III
NP-IV
NP-V
NP-VI

g MP

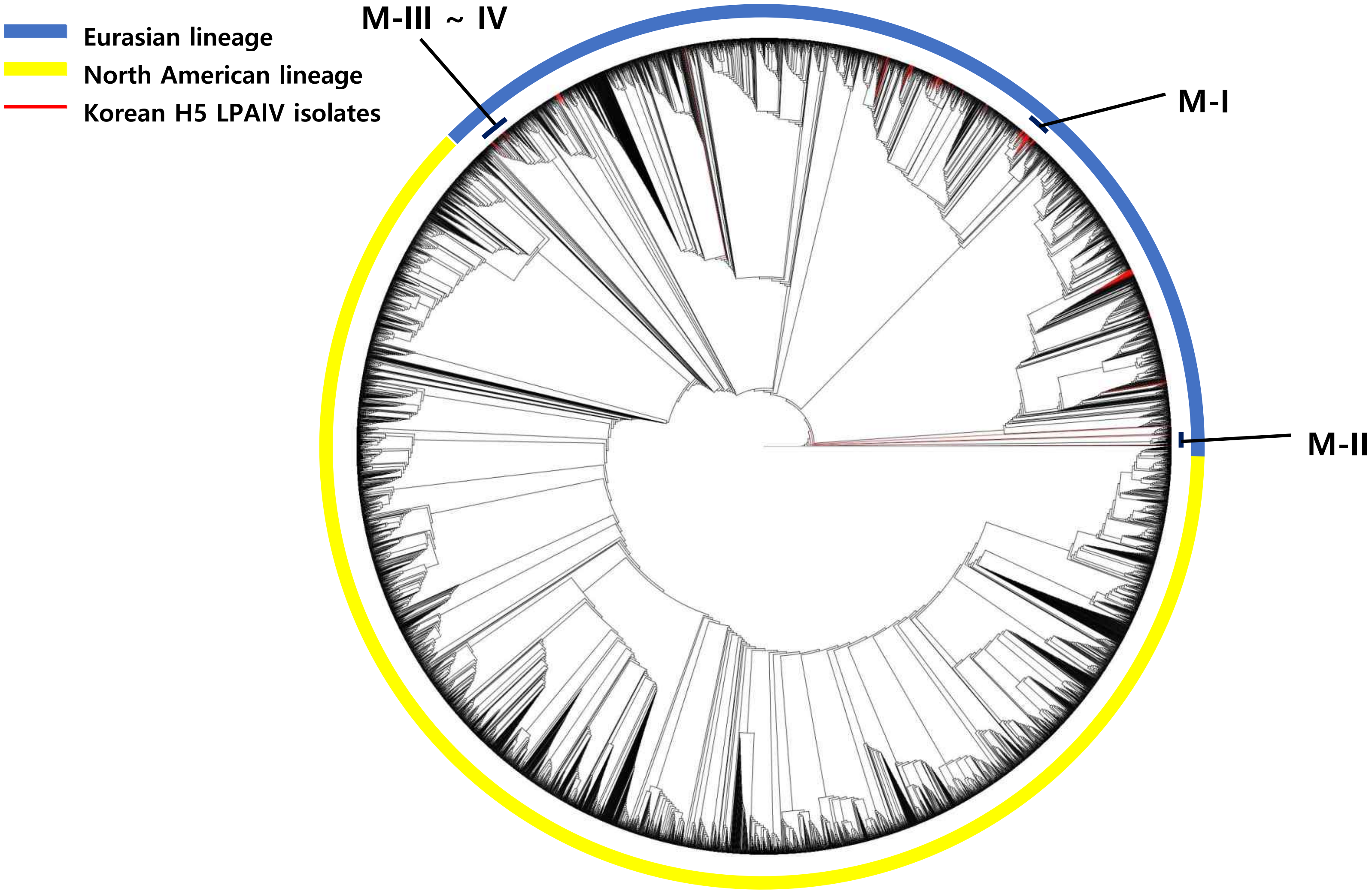

Eurasian lineage
North American lineage
Korean H5 LPAIV isolates
M-III ~ IV
M-I
M-II

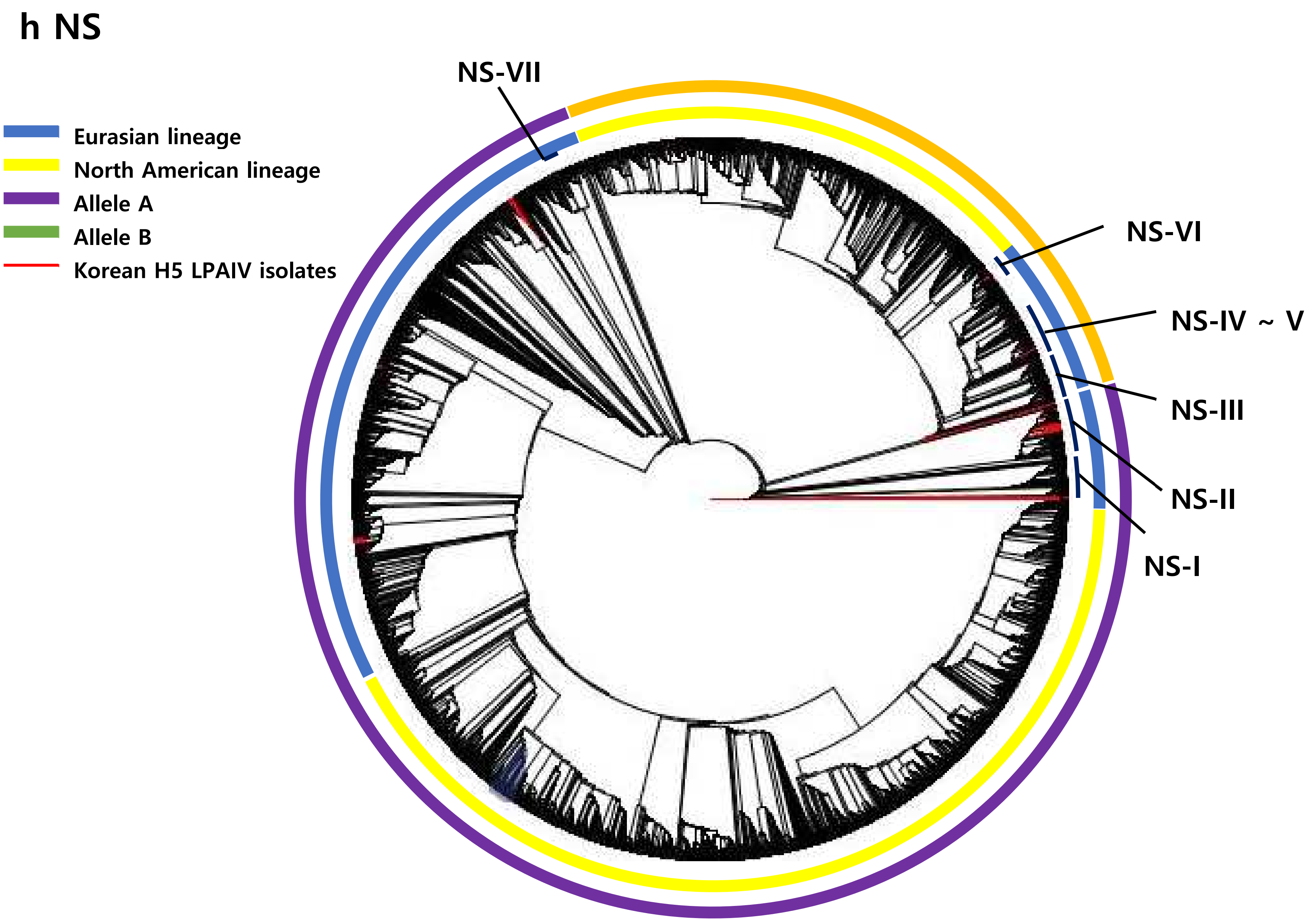
h NS
Eurasian lineage
North American lineage
Allele A
Allele B
Korean H5 LPAIV isolates
NS-VII
NS-VI
NS-IV ~ V
NS-III
NS-II
NS-I
